# The One Health High-Level Expert Panel (OHHLEP)

**DOI:** 10.1186/s42522-023-00085-2

**Published:** 2023-12-07

**Authors:** Thomas C. Mettenleiter, Wanda Markotter, Dominique F. Charron, Wiku B. Adisasmito, Salama Almuhairi, Casey Barton Behravesh, Pépé Bilivogui, Salome A. Bukachi, Natalia Casas, Natalia Cediel Becerra, Abhishek Chaudhary, Janice R. Ciacci Zanella, Andrew A. Cunningham, Osman Dar, Nitish Debnath, Baptiste Dungu, Elmoubasher Farag, George F. Gao, David T. S. Hayman, Margaret Khaitsa, Marion P. G. Koopmans, Catherine Machalaba, John S. Mackenzie, Serge Morand, Vyacheslav Smolenskiy, Lei Zhou

**Affiliations:** 1 Friedrich-Loeffler-Institut, Bundesforschungsinstitut Für Tiergesundheit, Federal Research Institute for Animal Health, Südufer 10, Greifswald, 17493 Insel Riems Germany; 2 Center for Viral Zoonoses, Department of Medical Virology, Faculty of Health Sciences, University of Pretoria, Pretoria, South Africa; 3 Visiting Professor, One Health Institute, University of Guelph, Guelph, ON Canada; 4 Universitas Indonesia, Depok, West Java Indonesia; 5 National Emergency Crisis and Disasters Management Authority, Abu Dhabi, United Arab Emirates; 6 Centers for Disease Control and Prevention, Atlanta, GA USA; 7 World Health Organization, Guinea Country Office, Conakry, Guinea; 8 Institute of Anthropology, Gender and African Studies, University of Nairobi, Nairobi, Kenia; 9 National Ministry of Health, Autonomous City of Buenos Aires, Argentina; 10 School of Agricultural Sciences, Universidad de la Salle, Bogotá, Colombia; 11 Indian Institute of Technology, Kanpur, India; 12 Brazilian Agricultural Research Corporation, Embrapa Swine and Poultry, Concórdia, Santa Catarina Brazil; 13 Institute of Zoology, Zoological Society of London, London, UK; 14 Global Operations Division, United Kingdom Health Security Agenca, London, UK; 15 Fleming Fund Country Grant to Bangladesh, DAI Global, Dhaka, Bangladesh; 16 Faculty of Veterinary Science, University of Kinshasa, Kinshasa, Democratic Republic of Congo; 17 Ministry of Public Health, Health Protection & Communicable Diseases Division, Doha, Qatar; 18 Institute of Microbiology, Chinese Academy of Sciences, Beijing, People’s Republic of China; 19 Molecular Epidemiology and Public Health Laboratory, Hopkirk Research Institute, Massey University, Palmerston North, New Zealand; 20 Mississippi State University, Starkville, MS USA; 21 Erasmus MC, Department of Viroscience, Rotterdam, The Netherlands; 22 EcoHealth Alliance, New York, NY USA; 23 Faculty of Health Sciences, Curtin University, Perth, Australia; 24 School of Chemistry and Molecular Biosciences, The University of Queensland, Brisbane, Australia; 25 HealthDEEP, CNRS, Montpellier, France; 26 Federal Service for Surveillance on Consumer Rights Protection and Human Well-being (Rospotrebnadzor), Moscow, Russian Federation; 27 Chinese Center for Disease Control and Prevention, Beijing, People’s Republic of China

## Introduction

One Health is an integrative and systemic approach to health, based on the understanding that human, animal and ecosystem health are inextricably linked. These interconnections and vulnerabilities were once more clearly demonstrated by the COVID-19 pandemic. This led the heads of the United Nations Food and Agriculture Organization (FAO), the United Nations Environment Programme (UNEP), the World Health Organization (WHO), and the World Organization for Animal Health (WOAH; founded as OIE), to enhance their science-based cross-sectoral collaboration by creating a multidisciplinary One Health High-Level Expert Panel (OHHLEP) to provide technical and scientific advice on One Health issues. Out of over 700 applications from all over the world, the four international partners FAO, WHO, WOAH and UNEP selected 26 experts from 24 countries as members of the OHHLEP. The multisectoral and transdisciplinary expertise present in OHHLEP members covers a wide range including animal, human and environmental health, biodiversity conservation and social sciences. The panel was conceived following a proposal by the French and German governments at the Paris Peace Forum in November 2020. It drew on the already existing FAO-OIE-WHO Tripartite intersectoral cooperation on One Health issues. In 2021, UNEP joined to form the Tripartite plus UNEP which was formally transformed into the ‘Quadripartite Collaboration for One Health’ in March 2022 and which now acts as the partner for engaging with OHHLEP. This is the first time that a global advisory panel on One Health has been created as a centre for expert advice. The OHHLEP convened for the first time on May 17, 2021 supported by a Secretariat that includes representation from each of the Quadripartite partners. WHO hosts the OHHLEP secretariat for the period 2021 to 2024, with this role rotating among the other partners in future years. At the inception meeting, Wanda Markotter and Thomas C. Mettenleiter were nominated as OHHLEP Co-Chairs and Dominique Charron was nominated Rapporteur. Biographies of OHHLEP members, reports of OHHLEP’s meetings, and related documents are available at https://www.who.int/groups/one-health-high-level-expert-panel/members.

OHHLEP has an advisory role to the Quadripartite partners and is expected to support their provision of evidence-based scientific and policy advice on One Health-related matters that support improved cooperation amongst governments to address the challenges raised by One Health. Subsequent to the COVID-19 pandemic, the initial term of OHHLEP focuses on zoonotic disease risk reduction and prevention. The areas of focus of OHHLEP are subject to regular review by the Quadripartite partners.

The terms of reference that were jointly developed by the Quadripartite partners specify that OHHLEP will “initially focus on: 1) providing policy relevant scientific assessment on the emergence of health crises arising from the human-animal-ecosystem interface, and research gaps; and 2) guidance on the development of a long term strategic approach to reducing risk of zoonotic pandemics, with an associated monitoring and early warning framework, and the synergies needed to institutionalize and implement the One Health approach, including in areas that drive pandemic risk”. Specifically, OHHLEP has been requested to perform the following functions.

- provide advice on the analysis of scientific evidence on the links between human, animal and ecosystem health, and contribute to foresight on emerging threats to health.
- provide advice on better understanding of the impacts of food systems (including agriculture, livestock farming and trade, wildlife hunting and trade, aquaculture, animal products, processing, handling, distribution and consumer practices) and ecological and environmental factors that may be contributing to zoonotic disease emergence/re-emergence and spillover events.
- contribute to the One Health research agenda setting and propose, advise on and review approaches and specific studies relevant to the development of a global approach to reducing the risk of pandemics of zoonotic origin.
- provide advice by invitation on One Health policy response in relevant member countries.
- provide recommendations on specific issues identified by the Partners in the areas of highest concern for attention and action, and future directions, in One Health.

OHHLEP also identified critical knowledge gaps on:

- the state of One Health implementation around the world.
- the lack of comprehensive databases and resources to support One Health implementation.
- a need for the mapping of existing initiatives, examples of success, and capacities for One Health research and implementation (One Health Workforce).
- the need for a model for an integrated One Health surveillance system and understanding of successful examples of existing One Health surveillance systems; consideration for how such a system could be used to detect previously unknown zoonotic diseases of public health importance.
- a more comprehensive understanding of the drivers of spillover of zoonotic diseases, and a standardized approach for assessing risks of spillover of pathogens between different animal populations and humans, and emergence of zoonotic diseases, including those arising in food systems.
- methodologies for identification and control of zoonotic pathogen spillover risks and for the spread of zoonotic diseases.

OHHLEP initially organized four working groups with dedicated participation of specific OHHLEP members that were later transformed into thematic groups open to all interested panelists to address the following workplan:

## One health implementation

- Define One Health in the context of the panel (see Output below).
- Develop a theory of change to convert One Health from a theoretical concept to the daily practice of collaborative work between the different sectors (health, agriculture, environment) and at different levels (see Output below).
- Identify technical and institutional barriers for implementation of One Health on the ground.
- Identify case studies demonstrating good practice in One Health in detecting, controlling and preventing emerging zoonoses. Specifically, what worked, how were barriers overcome, governance arrangements, funding, incentives, etc.
- Suggest/develop improved flexible implementation strategies for One Health, focusing on preventing emerging zoonoses in different contexts.

## Inventory of current knowledge in preventing emerging zoonoses

- Systematic review and inventory of useful documents, knowledge-sharing platforms, capacity building tools, projects, networks, committees and good practices for the use of One Health approaches in the prevention of emerging zoonoses (currently ongoing)
- Identify successful transnational and national strategies and/or ministerial/administrative set-up that have shown practical and useful intersectoral collaboration (examples of good practice; currently ongoing)
- Review requirements for a One Health workforce
- Develop and prioritize a portfolio of issues that would make a difference in the prevention of emerging zoonoses at the global, regional and national level

## Develop a One Health Framework for surveillance, early detection, and rapid data sharing in the prevention of emerging zoonoses

- Define the model One Health surveillance system (see Output below)
- Develop a practically implementable surveillance framework and good practice guidelines (currently ongoing)
- Assess what is known today about the presence of potential zoonotic pathogens, including current hotspot identification work
- Identify existing international guidance for integrated disease surveillance and the level of implementation
- Identify existing agreements and systems allowing/facilitating sharing of surveillance data.
- Provide guidance for interlaboratory systems for sharing of samples, data, results to provide early detection and diagnosis of disease pathogens

## Identify factors contributing to spillover and subsequent spread of diseases and develop risk management framework

- Identify key drivers of spillover (currently ongoing) including factors such as wildlife trade, food production and distribution, traditional markets, land-use changes, biodiversity, animal production and trade, human action, biosafety and biosecurity, any other relevant environmental issues, including climate change
- Consider tools already available for multisectoral risk assessments; for example investigate if HACCP principles could be adapted as risk assessment possibilities.
- Systematically analyze the evidence for zoonotic spillover risk.
- Identify the knowledge gaps and the factors that are neglected and what should be prioritized.

## OHHLEP Outputs

### One Health Definition

OHHLEP’s first deliverable was an inclusive and expanded definition of One Health [1], which was endorsed by the Quadripartite partners and accepted globally. The One Health definition developed by the OHHLEP states:

- One Health is an integrated, unifying approach that aims to sustainably balance and optimize the health of people, animals and ecosystems.
- It recognizes the health of humans, domestic and wild animals, plants, and the wider environment (including ecosystems) are closely linked and interdependent.
- The approach mobilizes multiple sectors, disciplines and communities at varying levels of society to work together to foster well-being and tackle threats to health and ecosystems, while addressing the collective need for healthy food, water, energy, and air, taking action on climate change, and contributing to sustainable development.

**Fig. 1:**
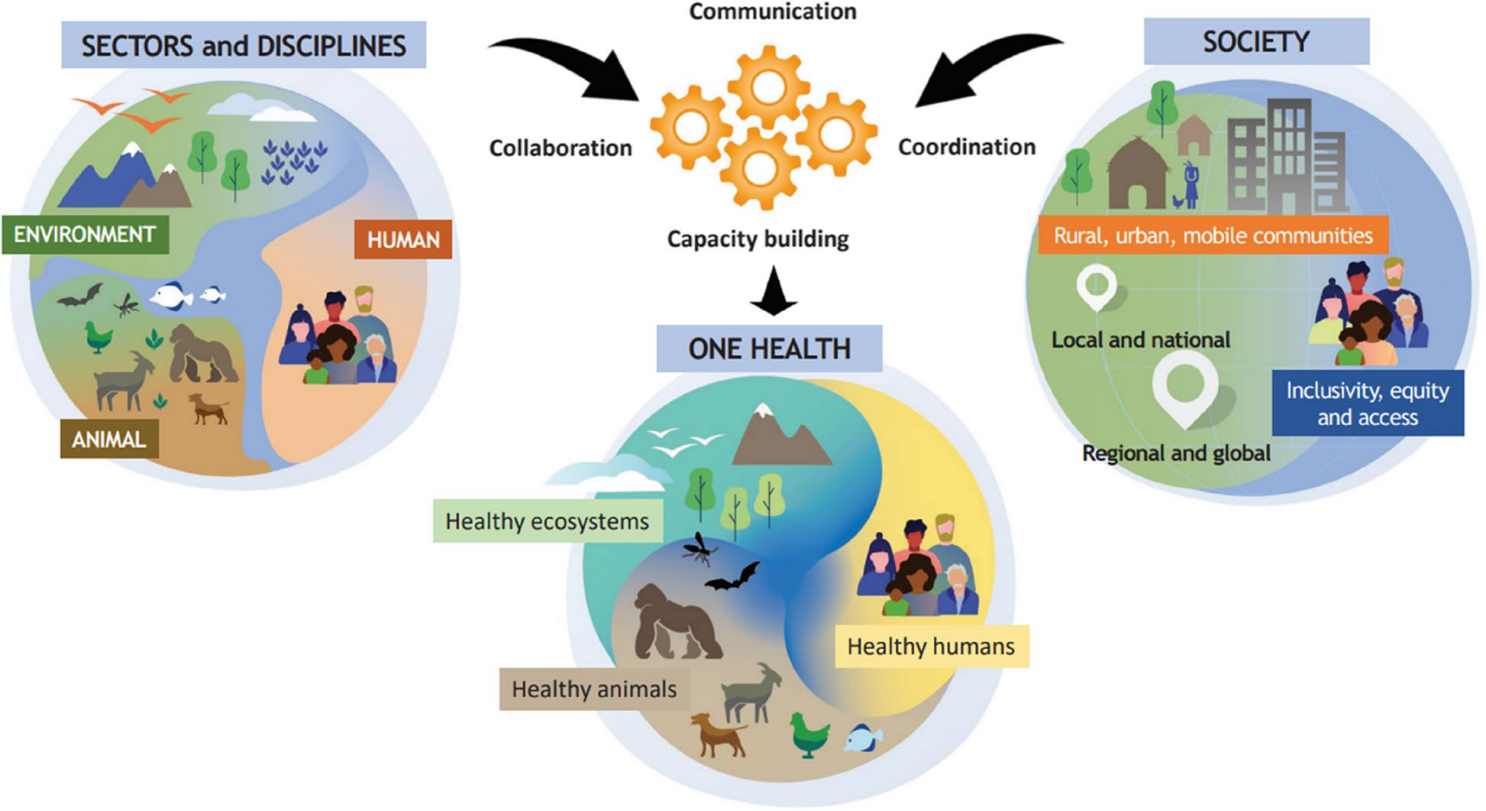
OHHLEP One Health definition visual [1]

To arrive at this definition, OHHLEP conducted a review of existing definitions used by the Quadripartite and other leading organizations from around the world. The definition builds on these and similar concepts from related fields of EcoHealth and Planetary Health. In addition to reflecting the interdependent health of people, animals and ecosystems as with most definitions of One Health, OHHLEP’s definition addresses intersectoral implementation considerations: propelling One Health from theory to practice, by highlighting the central role of intersectoral actions with the ‘4C’s: communication, coordination, collaboration and capacity building (Fig. 1) and has foundational principles that ensure One Health actions are effective, fair, equitable and sustainable.

The OHHLEP One Health definition is comprehensive and promotes a clear understanding and holistic approach across sectors and areas of expertise. While health, food, water, energy, and environment are all wider topics with sector-specific and specialist concerns beyond the scope of One Health approaches, their interdependence highlights where multiple sectors have shared responsibility and relevance in protecting health, addressing health challenges such as the emergence of infectious diseases and promoting the health and integrity of our ecosystems. The One Health approach can potentially address the full spectrum of infectious and non-infectious disease control from prevention, health improvement and health promotion, to the detection, preparedness for, response and recovery from health crises.

The approach is applicable at community, subnational, national, regional, and global levels, and relies on demonstrating the added value of shared and effective governance, communication, collaboration and coordination to understand co-benefits, cost-efficiency, risks, trade-offs and opportunities for equitable and holistic solutions.

Additionally, while the scope of OHHLEP’s work relates strongly to the broad aims and objectives of health security (as well as by extension, sustainable food, water and energy security), we wish to strike a careful balance between the hard realities of a global geopolitical paradigm dominated by economics, security and self-interest, and our collective aspirations for a better world.

The OHHLEP One Health definition is accompanied by foundational principles that help to ensure One Health actions are effective, fair, equitable and sustainable.

- Equity between sectors and disciplines.
- Sociopolitical and multicultural parity (the doctrine that all people are equal and deserve equal rights and opportunities) and inclusion and engagement of communities and marginalized voices.
- Socio-ecological equilibrium that seeks a harmonious balance between human—animal-environment interaction and acknowledging the importance of biodiversity, access to sufficient natural space and resources, and the intrinsic value of all living things within the ecosystem.
- Stewardship and the responsibility of humans to change behaviour and adopt sustainable solutions that recognize the importance of animal welfare and the integrity of the whole ecosystem, thus securing the well-being of current and future generations.
- Transdisciplinarity and multisectoral collaboration which includes all relevant disciplines, both modern and traditional forms of knowledge and a broad representative array of perspectives.

### Quadripartite One Health Joint Plan of Action

OHHLEP provided input into the One Health Joint Plan of Action (JPA; [2]), a strategic document by the Quadripartite outlining the way forward for these partners’ successful implementation of the One Health approach to tackle global problems at the human-animal-ecosystem interface. This also aligns with key needs to achieve the United Nations sustainable development goals, and as guiding principles for policy makers, scientists and practitioners alike.

A major contribution of OHHLEP was to the development of the JPA’s Theory of Change [2]. This was an example of OHHLEP members collaborating closely with partners on a knowledge product and overarching strategy, to help shape this first collective Quadripartite document that will be used to guide the actions and activities of the Quadripartite organizations into the future. With a 5-year horizon, the OH JPA aims to be a technical document providing a framework with joint vision and commitment allowing the four partner organizations to work together effectively to implement a One Health approach. Through the OH JPA, the partners also aim to support One Health implementation by member countries, enable collaboration across sectors and regions, identify synergies and overlaps to support coordination and mobilize investment including better use of resources.

The two OH JPA long-term outcomes are the development of: (i) Improved health of humans, animals, plants and the environment while identifying sustainable system-wide One Health solutions that allow our ecosystems to thrive in harmony; (ii) Reduced risk and impact of health threats at the human-animal-plant-environment interface using a One Health approach efficiently, effectively, and equitably.

It describes medium-term outcomes for the period 2022–2026, that will be achieved by implementing actions along three Pathways of change:

- Pathway 1 **–** Governance, policy, legislation, financing, and advocacy
- Pathway 2** –** Organizational & institutional development, implementation, and sectoral integration
- Pathway 3 **–** Data, evidence, information systems, and knowledge exchange

The JPA has 6 interdependent Action Tracks with associated detailed lists of activities, deliverables and timeline with one being overarching on strengthening One Health systems. The six OH JPA Action Tracks are focused on:

- Enhancing One Health capacities to strengthen health systems
- Reducing the risk of Emerging zoonotic epidemics and pandemics
- Controlling and eliminating Endemic zoonotic, neglected tropical and vector-borne diseases
- Strengthening the assessment, management and communication of Food safety risks
- Curbing the silent pandemic of antimicrobial resistance
- Integrating the environment into One Health

In addition to supporting the production of a global vision and roadmap for One Health, OHHLEP’s collaboration in the development of the OH JPA helped to proactively identify where OHHLEP may best be able to contribute to the pursuit of target outputs and outcomes. It also helped to clarify for OHHLEP the parameters of the plan, including aspects outside of the scope of the OH JPA where partners additional to the four organizations may be relevant when developing OHHLEP’s Theory of Change. As of early 2023, the Panel has been providing inputs into the development of the implementation guide of the OH JPA.

### OHHLEP Theory of Change (ToC)

While developing the working definition of One Health, OHHLEP initiated a process of drafting its own ToC for One Health [3, 4]. Existing ToC frameworks used at national and organizational level were consulted to learn from and build on prior processes. The timing of this initial work was ideal to inform the Quadripartite JPA ToC. Accordingly, the ToC’s of OHHLEP and the Quadripartite Partners are closely aligned. The Quadripartite OH JPA is targeted to specific action tracks for which the international partners will be responsible. OHHLEP’s efforts and its One Health ToC have flexibility in scope, for example to include community-based considerations and actions more directly. The OHHLEP ToC also identifies the activities to be pursued by OHHLEP specifically, while also connecting with the Quadripartite partners’ ToC to reach target outcomes.

Both ToC include three central pathways of change. The OHHLEP ToC also includes a problem statement that identifies over 60 societal, animal and environmental challenges stemming from inter-linked categories of human activity (anthropogenic influences on health) and an overview of relevant actors, in addition to its pathways of change, barriers and enabling factors, activities, outcomes and impacts.

The OHHLEP ToC [3, 4] is envisioned as a working document, with the expectation that it will be reviewed periodically and updated as the needs and landscape related to One Health evolve at global, national, regional, and subnational levels.

### Inventory of One Health Resources

For OHHLEP to understand and advise the Quadripartite partners in the One Health sphere, it was necessary to create an inventory of relevant partner initiatives and other relevant activities and initiatives globally. The plethora of new initiatives labelled as One Health makes it difficult to systematically keep track and evaluate the current scope and coverage of efforts. OHHLEP first obtained information from the Quadripartite partners regarding their initiatives. OHHLEP also started collating information more broadly. An initial database has been created and populated by all OHHLEP members and Quadripartite partners on One Health tools, guidance, frameworks, and relevant documents for an updated analysis of these tools for capacity assessment and operationalization to ensure optimal outcomes for countries and regions using available One Health tools, to provide an updated framework to support implementation, to identify gaps and priorities for the development of additional tolls including the environmental dimension, and to provide guidance on coordination and sharing of outputs. Details of the focus and scope of One Health or definition used were included to assess the extent to which the One Health concept was applied. The specific geographic region and engaged sectors are also included. OHHLEP also initiated an inventory of published literature on One Health relating to emerging zoonotic disease threats.

### Develop a model surveillance system

The definition, scope and purpose of surveillance differs across sectors, objectives, and contexts. Given the new OHHLEP One Health definition and the historically siloed surveillance systems across the human, animal and ecosystem health dimensions of One Health, defining an optimal health surveillance system that is operationally feasible was a key goal of OHHLEP.

OHHLEP reviewed what is currently considered as One Health surveillance, compiled examples of existing One Health surveillance systems, and identified key components of an optimal One Health surveillance system. This was done using inventories of the Quadripartite partners, literature review, and questionnaires completed by OHHLEP members. These indicated that there are clear gaps in surveillance that, often for reasons outside of the scope of the partners, due to previous historic priorities, are not dealt with by any agency. This is also reflected in national surveillance systems.

Key elements of a model One Health surveillance system were described, focusing on leadership, communication and coordination, and operational implementation. Although primarily intended for international partners, the OHHLEP One Health surveillance framework could also be implemented at the regional, national or local level. Key elements of the system were identified including:

- Strong governance and accountability of One Health functions and policies; high-level leadership.
- Multisectoral: public health and medicine, animal health and environment were all considered equally important.
- Independent scientific expert advice to the decision makers.
- Coordination office and functions across sectors and across jurisdictions and internationally; consideration of nesting similar coordination and roles for One Health at all levels.
- Cooperation and coordination across laboratory, clinical, public and animal health agencies and environmental monitoring agencies.
- Consideration to be given on the role of other sectors such as finance, public safety in supporting One Health surveillance; role of non-governmental stakeholders such as research and academic institutes, community and civil society organizations.
- Implementation challenge is considerable; barriers and knowledge gaps were identified.
- Although most available examples focus on detection of human and zoonotic animal pathogens, there is a need to consider integrating environmental and socio-economic drivers of spillover.

The first results have been submitted for publication to inform on a best practice model system for One Health surveillance (One Health, in press).

### Identify drivers of zoonotic spillover and risk assessment

Increasing evidence suggests that the majority of novel, emerging infectious diseases of humans originate from animals, and of those, that the majority spilled over from wild animals over recent decades. Major drivers of this emergence are human activities, including land use and ecosystem changes, and changes to the ways people interact with animals, such as new or increased human-wildlife interfaces. Most of these, in turn, have complex social, cultural and economic drivers. Traditionally, and even after the emergence of COVID-19, the response to disease emergence and spread has been to focus on increasing human knowledge of what pathogens exist, where they may be found, and improving early detection and surveillance of cases of human disease. Unfortunately, these approaches do not prevent zoonotic spillover events and as exemplified by COVID-19, even relatively early detection of a new disease does not necessarily mean it will be effectively contained. OHHLEP seeks to collate an evidence base to more precisely identify the upstream drivers of zoonotic spill-over, and how to mitigate these to prevent disease emergence from occurring in the first place.

OHHLEP began work to identify key drivers of zoonotic disease spillover, drawing on expert opinion which was then used to inform an extensive review of the literature. Based on the findings from the literature review, a risk assessment and critical control point framework will be tried for the drivers associated with zoonotic disease emergence. Eleven putative anthropogenic upstream drivers were initially identified.

These identified anthropogenic drivers increase the interface and interaction between humans, wildlife and domestic animals. They include practices related to wildlife hunting, capture and consumption; wildlife framing and trade; unsustainable agricultural practices for livestock and crops production; climate change, urbanization and the fragmentation of natural habitats. Given the diversity of published evidence for the role and importance of drivers of zoonotic spillover, OHHLEP initiated a systematic review. These eleven identified drivers are currently being assessed first through a systematic review of reviews of the evidence for drivers of zoonotic spillover with a draft summary due in mid-2023.

### Prevention of zoonotic spillover white paper

The Quadripartite asked OHHLEP to prepare a white paper on the Definition of Prevention of Zoonotic Spillover [5] to inform the discussions around the forthcoming global Pandemic Instrument being negotiated by World Health Assembly member states. The purpose of the white paper was to highlight the importance of spillover prevention within the triad of prevention, preparedness, and response. The paper provides a definition of this scope of prevention and points out that strategies to reduce the probability of spillover events are under-prioritised and under-utilized, and have been lost in overall preparedness discussions and recovery financing. A disproportionate focus on detection and response suggests that a number of factors, such as insufficient evidence, complex mechanisms, and lack of political will lead to the allocation of attention and financial resources to infectious disease problems only once they have occurred, rather than taking the steps necessary to reduce the risk of their occurrence in the first place. The paper argues that addressing the drivers of pathogen spillover through a One Health approach has significant subsequent economic and social co-benefits. The white paper was published on the OHHLEP website in early 2023, and has been shared with the Intergovernmental Negotiating Body to inform discussions on the Pandemic Instrument. It has recently been submitted to a scientific journal PLoS Pathogens, in press.

### Other OHHLEP activities

The Quadripartite invited OHHLEP to provide input to the zero draft of the Pandemic Instrument, to strengthen considerations of prevention and a One Health approach to pandemic preparedness and response. Recently OHHLEP was asked to present during a member state consultation on the Zero draft and the Quadripartite will continue to facilitate OHHLEP input to subsequent drafts.

OHHLEP has also engaged with the Pandemic Fund hosted by the World Bank, sharing its Prevention White Paper and engaging in informal discussions around the placement of prevention within the call for proposals. Some OHHLEP members also serve on the Governing Board and Technical Advisory Panel of the Pandemic Fund. OHHLEP has also been requested to jointly host a meeting with the World Bank and the Quadripartite partners to discuss coordination of methodologies for One Health Assessment in view of applications to the Pandemic Fund and the roll out of the OH Joint Plan of Action in countries and with the aim of streamlining and making the exercise more efficient for countries.

Since its inception, OHHLEP co-chairs and members sought to build connections toward collaboration with other key One Health global initiatives, e.g., the WHO Scientific Advisory Group for the Origins of Novel Pathogens (SAGO), the Quadripartite Global Leaders Group on Antimicrobial Resistance (GLG-AMR), and the One Health Intelligence Scoping Study (OHISS), among others. They were also active in numerous national and international engagements to promote One Health approaches.

## Conclusion and next steps

As the COVID-19 pandemic has drastically demonstrated, there remains an urgent need for greater multisectoral and multilateral collaboration at all levels to help end the pandemic and for better prevention of as well as improved preparedness for future pandemics and other health threats that arise at the animal-human–environment interface.

The threats to the health of humans, animals and ecosystems are increasingly apparent and growing, due to combined crises of climate change and declining biodiversity, among other pressures on our collective health and wellbeing. Therefore, the work of OHHLEP continues to be very timely and relevant. Thanks to fast action by the Quadripartite partners, OHHLEP was convened and began its work within a short period of time. With its input into the development of the Quadripartite One Health Joint Plan of Action, OHHLEP has shown its capacity to support policy development in real time. The publication of OHHLEP’s definition of One Health has shown its ability to work rapidly to contribute ideas that catalyze dialogue and inform OH implementation by the Quadripartite partners and other stakeholders. Much work has also been done by OHHLEP towards developing an inventory of One Health resources, creating a model integrated One Health surveillance system, identifying key drivers of zoonotic disease spillover and developing a model risk assessment and critical control point framework. None of this would have been possible without the support and genuine collaboration of experts from across all Quadripartite partners in a true spirit of One Health cooperation. Going forward in its second term, OHHLEP will pursue its work to advice the Quadripartite partners in collaboration and with the support of their numerous initiatives and structures, and, increasingly, in collaboration with other relevant international and regional One Health initiatives.
