# Correction: The One Health High-Level Expert Panel (OHHLEP)

**DOI:** 10.1186/s42522-024-00096-7

**Published:** 2024-03-27

**Authors:** Thomas C. Mettenleiter, Wanda Markotter, Dominique F. Charron, Wiku B. Adisasmito, Salama Almuhairi, Casey Barton Behravesh, Pépé Bilivogui, Salome A. Bukachi, Natalia Casas, Natalia Cediel Becerra, Abhishek Chaudhary, Janice R. Ciacci Zanella, Andrew A. Cunningham, Osman Dar, Nitish Debnath, Baptiste Dungu, Elmoubasher Farag, George F. Gao, David T. S. Hayman, Margaret Khaitsa, Marion P. G. Koopmans, Catherine Machalaba, John S. Mackenzie, Serge Morand, Vyacheslav Smolenskiy, Lei Zhou

**Affiliations:** 1 Friedrich-Loeffler-Institut, Bundesforschungsinstitut für Tiergesundheit, Federal Research Institute for Animal Health, Südufer 10, 17493 Greifswald, Insel Riems Germany; 2 Center for Viral Zoonoses, Department of Medical Virology, Faculty of Health Sciences, University of Pretoria, Pretoria, South Africa; 3 One Health Institute, University of Guelph, Guelph, ON Canada; 4 Universitas Indonesia, Depok, West Java Indonesia; 5 National Emergency Crisis and Disasters Management Authority, Abu Dhabi, United Arab Emirates; 6 Centers for Disease Control and Prevention, Atlanta, GA USA; 7 World Health Organization, Guinea Country Office, Conakry, Guinea; 8 Institute of Anthropology, Gender and African Studies, University of Nairobi, Nairobi, Kenya; 9 National Ministry of Health, Autonomous City of Buenos Aires, Argentina; 10 School of Agricultural Sciences, Universidad de la Salle, Bogotá, Colombia; 11 Indian Institute of Technology, Kanpur, India; 12 Brazilian Agricultural Research Corporation, Embrapa Swine and Poultry, Concórdia, Santa Catarina Brazil; 13 Institute of Zoology, Zoological Society of London, London, UK; 14 Global Operations Division, United Kingdom Health Security Agency, London, UK; 15 Fleming Fund Country Grant to Bangladesh, DAI Global, Dhaka, Bangladesh; 16 Faculty of Veterinary Science, University of Kinshasa, Kinshasa, Democratic Republic of Congo; 17 Ministry of Public Health, Health Protection & Communicable Diseases Division, Doha, Qatar; 18 Institute of Microbiology, Chinese Academy of Sciences, Beijing, People’s Republic of China; 19 Molecular Epidemiology and Public Health Laboratory, Hopkirk Research Institute, Massey University, Palmerston North, New Zealand; 20 Mississippi State University, Starkville, MS USA; 21 Erasmus MC, Department of Viroscience, Rotterdam, The Netherlands; 22 EcoHealth Alliance, New York, NY USA; 23 Faculty of Health Sciences, Curtin University, Perth, Australia; 24 School of Chemistry and Molecular Biosciences, The University of Queensland, Brisbane, Australia; 25 HealthDEEP, CNRS, Montpellier, France; 26 Federal Service for Surveillance on Consumer Rights Protection and Human Well-being (Rospotrebnadzor), Moscow, Russian Federation; 27 Chinese Center for Disease Control and Prevention, Beijing, People’s Republic of China

**Correction to: One Health Outlook (2023) 5:18**

10.1186/s42522-023-00085-2

After publication of this article, it was reported that in section ‘OHHLEP Outputs, One Health Definition’, the last line of the One Health definition, ‘The approach mobilizes multiple sectors, disciplines and communities at varying levels of society to work together to foster well-being and tackle threats to health and ecosystems, while addressing the collective need for clean water, energy and air, safe and nutritious food, taking action on climate change, and contributing to sustainable development.’ should have read The approach mobilizes multiple sectors, disciplines and communities at varying levels of society to work together to foster well-being and tackle threats to health and ecosystems, while addressing the collective need for healthy food, clean water, energy, and air, taking action on climate change, and contributing to sustainable development.’

In the section ‘Quadripartite One Health Joint Plan of Action’, the sixth bullet point of the JPA action tracks, ‘Integrating the into One Health’ should have read ‘Integrating the environment into One Health’.

In the Acknowledgements section, the affiliation of Chadia Wannous should have read ‘WOAH’ instead of ‘WHO’.

The original article has been corrected.

